# Rationally Designed TadA‐Derived Cytosine Editors Enable Context‐Independent Zebrafish Genome Editing

**DOI:** 10.1002/advs.202509800

**Published:** 2025-07-20

**Authors:** Wei Qin, Sheng‐Jia Lin, Yu Zhang, Kevin Huang, Cassidy Petree, Kevin Boyd, Pratishtha Varshney, Gaurav K. Varshney

**Affiliations:** ^1^ Genes & Human Disease Research Program Oklahoma Medical Research Foundation Oklahoma City Oklahoma 73104 USA; ^2^ Cell & Cancer Biology Research Program Oklahoma Medical Research Foundation Oklahoma City Oklahoma 73104 USA

**Keywords:** cytosine base editors, disease models, genome editing, zebrafish

## Abstract

CRISPR base editors are crucial for precise genome manipulation. Existing APOBEC‐based cytosine base editors (CBEs), while powerful, exhibit indels and sequence context limitations, and editing CC and GC motifs is challenging and inefficient. To address these challenges, existing tRNA adenine deaminase (TadA)‐derived CBEs are evaluated in zebrafish, and a series of zTadCBE variants is developed that demonstrate high editing efficiency, minimized off‐target effects, and an expanded targeting range compared to existing tools. The approach integrates beneficial mutations from TadA‐based adenine base editors (ABEs) with SpRYCas9n‐enhanced protospacer‐adjacent motif (PAM) compatibility. The expanded window zTadCBE variants enable the targeting of cytosines at a broader range of nucleotide positions relative to the PAM sequence, further enhancing the versatility of this tool. Using zTadCBEs, four zebrafish disease models affecting the auditory, nervous, metabolic, and muscular systems are generated directly in the F0 generation—models that cannot be efficiently produced using earlier CBE tools. Together, zTadCBE variants provide a robust and flexible toolkit for efficient and precise C‐to‐T base editing in zebrafish, facilitating rapid in vivo functional assessment of genetic variants.

## Graphical abstract

A next‐generation cytosine base editor (zTadCBE) and other variants are developed by combining the high‐efficiency (TadCBEa) and low‐indel (TadCBEMax) architectures, followed by the introduction of zebrafish‐optimized mutations (V82S, Q154R) in the TadA domain. This optimized tool enables precise modeling of hearing, neurological, metabolic, and muscular disorders in zebrafish.

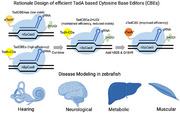

## 1 Introduction

Genome editing has experienced a remarkable breakthrough with the development of clustered regularly interspaced short palindromic repeat (CRISPR) base editors, enabling the precise and efficient modification of single nucleotides in the genome without introducing double‐stranded breaks. This technology offers powerful capabilities for studying genetic variants in cell lines and animal models, effectively mimicking certain human genetic diseases or variants of unknown significance (VUS). Base editors comprise a catalytically inactive CRISPR‐associated protein 9 (Cas9) fused to a deaminase enzyme and fall into two primary classes: cytosine base editors (CBEs) and adenine base editors (ABEs). CBEs use a cytidine deaminase such as rat APOBEC1 (rAPOBEC‐1) that converts cytosine (C) to uracil (U), which can be recognized and excised by uracil DNA glycosylase (UDG), thereby initiating the base excision repair (BER) pathway. This repair process not only diminishes the efficiency of the intended C‐to‐T conversion but may also generate undesired byproducts such as abasic sites, strand breaks, and indels. The inclusion of uracil glycosylase inhibitor (UGI) inhibits UDG activity, thereby preventing uracil excision and allowing the replication machinery to interpret uracil as thymine, leading to a more efficient and stable C‐to‐T substitution.^[^ ^1^ ^]^ On the other hand, ABEs utilize a laboratory‐evolved tRNA adenine deaminase (TadA) that specializes in converting adenine (A) to guanine (G), or its complementary base thymine (T) to cytosine (C).^[^ ^2^ ^]^

Several CBEs, such as BE3, Target‐AID, BE4‐Gam, BE4max, and AncBE4max, have been tested in cultured cells, plants, and various animal models.^[^ ^2^, ^3^, ^4^, ^5^, ^6^, ^7^ ^]^ While they offer great promise for genome editing in some systems, they show variable or limited editing efficiencies, substantial off‐target editing, and induce significant insertions and deletions (indels) in zebrafish and many other model organisms.^[^ ^8^, ^9^, ^10^ ^]^ Even the most advanced and efficient CBE – AncBE4max, which harbors an ancAPOBEC1 deaminase domain,^[^ ^1^, ^2^, ^7^, ^8^, ^11^, ^12^ ^]^ suffers from sequence context preferences that limit the genomic sites it can target.

Many groups have optimized CBEs using different cytosine deaminases and engineered variants (e.g., hAPOBEC3A, PmCDA1, hAID, and Anc689) to reduce off‐target DNA edits and broaden editing scope.^[^ ^7^, ^13^, ^14^, ^15^ ^]^ For example, the improved editing efficiency of AncBE4max comes from the use of modified nuclear localization sequences (NLSs) to enhance its nuclear entry, as well as a codon‐optimized version of the ancestral deaminase Anc689 APOBEC.^[^ ^7^ ^]^

In contrast to cytidine deaminases, the TadA enzymes used by ABEs are relatively less processive and induce A‐to‐G DNA conversions with over 99.9% purity, minimal indels, and a compact editing window. These properties allow current state‐of‐the‐art ABE variants, like ABE8e (also called TadA‐8e) or our high‐efficiency variant ABE‐ultramax,^[^ ^16^ ^]^ to achieve higher editing efficiencies—up to 100% as well as greater single‐nucleotide precision and lower Cas‐independent off‐target editing than CBEs.^[^ ^17^ ^]^ New research has therefore focused on re‐engineering the TadA enzyme from TadA‐8e to produce artificial variants that deaminate cytosine instead of adenine, overcoming the challenges posed by CBEs.^[^ ^18^, ^19^, ^20^ ^]^ Based on TadA engineering, multiple variants of Tad‐CBEs, such as TadCBEa, TadCBEd, TadCBEd_V106W, TadDE, eTd‐CBE, Td‐CBEmax, and CBE‐T1.14, have been generated that can mediate efficient C‐to‐T conversions in human cells and plants.^[^ ^18^, ^19^, ^20^, ^21^, ^22^ ^]^

However, the application of TadA variants in zebrafish, a key model system for studying human genetic diseases, due to its genetic versatility, rapid development, and similarities to mammals remains limited. Successful demonstrations of CBEs are restricted to a few loci, with only zAncBE4‐max and BE4‐Gam tested with variable efficiencies,^[^ ^3^, ^8^, ^11^, ^23^ ^]^ leaving the potential of these variants for optimizing zebrafish CBEs largely unexplored.

Here, we comprehensively evaluated three TadA‐derived variants for C‐to‐T editing in zebrafish. Our analysis revealed that TadCBEa mediates highly efficient C‐to‐T editing, while TadCBEmax induces the fewest indels. Building on these findings, we developed a novel TadA‐derived CBE, zTadCBE, which combines high editing efficiency with low indel rates. Our application of this tool to generate zebrafish disease models further underscored its practical utility, enabling the targeting of previously inaccessible loci. These results highlight the significant potential of zTadCBE for expanding the scope of functional genomics and disease modeling in zebrafish.

## 2 Results

### 2.1 Evaluation and Comparison of the In Vivo Editing Efficiencies of Three Representative TadA‐Derived CBEs in Zebrafish

**Figure 1:**
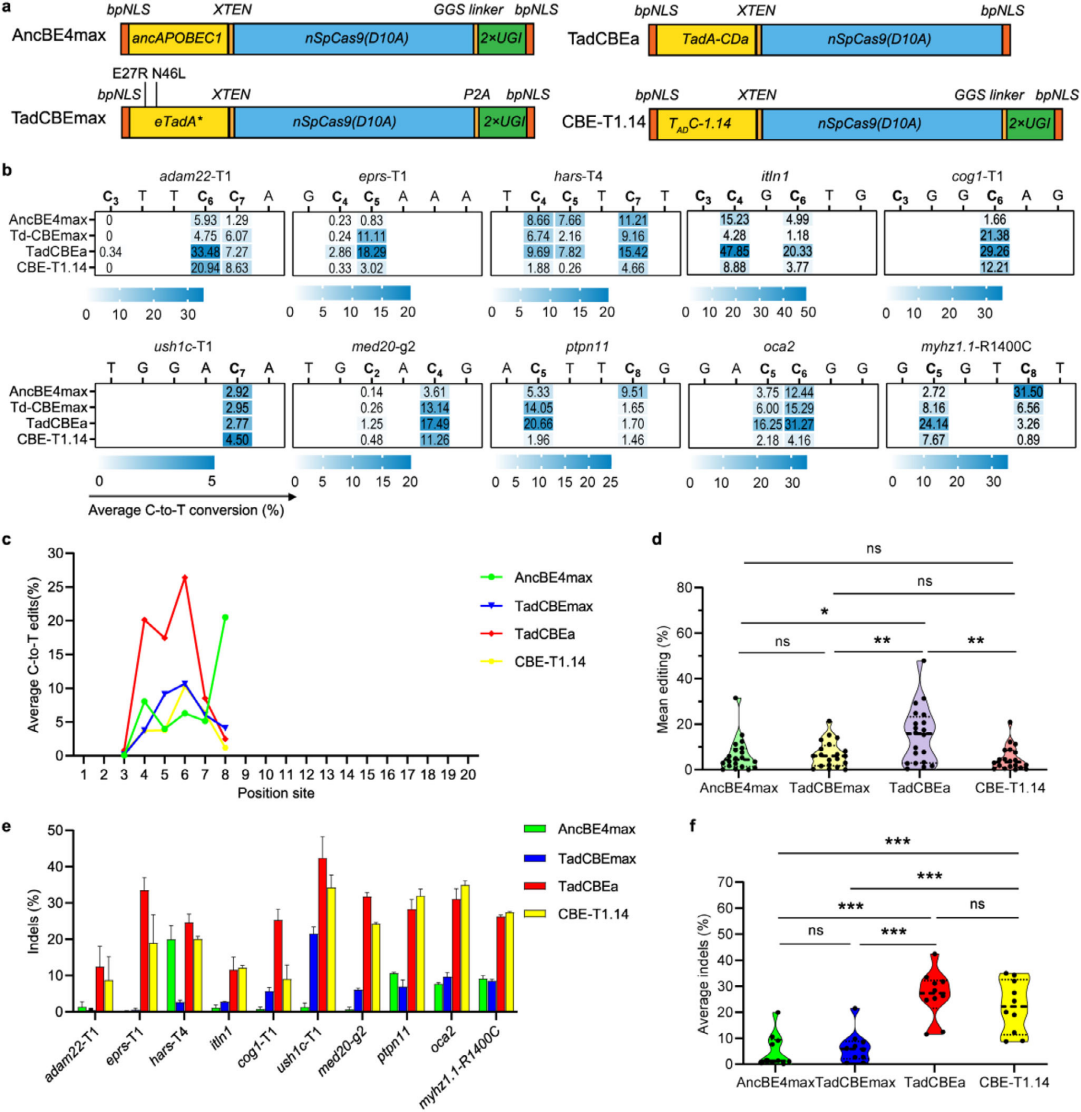
Comparative evaluation of in vivo editing efficiencies for three representative TadA‐derived cytosine base editors in zebrafish. a) Schematic of the mRNA construct for four cytosine base editors. bpNLS: bipartite nuclear localization, apobec1, eTadA*, TadA‐CDa and CBE‐1.14: various adenine deaminase, XTEN: a 32aa flexible linker, nSpCas9: SpCas9 nickase, GGS linker: GGSSGGS amino acid, P2A: Porcine teschovirus‐1 2A, UGI: Uracil glycosylase inhibitor. b) The C‐to‐T editing efficiency of AncBE4max, TadCBEmax, TadCBEa, CBE‐1.14 was examined at 10 endogenous genomic loci. The heatmap represents the average editing percentage derived from three independent experiments. c) Evaluation of the efficiency and targeting window of all four CBEs based on 10 sites in Figure 1b. Each data point reflects the mean editing activity at a specific site (PAM located at positions 21–23). Data from three independent experiments were analyzed. d) Assessment of the mean editing efficiency of four cytosine base editors using the plot based on the data in Figure 1b. Mean editing efficiency at each site is represented by individual data points, with the central dotted line indicating the overall mean. Two‐tailed paired t‐test was performed: not significant (ns) *P* ≥0.05, * *P* < 0.05, ** *P* < 0.01, and *** *P* < 0.001. e) The indel efficiency comparison among four cytosine base editors targeting ten different loci. Values are presented as mean value ± standard deviation (SD), n = 3 biological replicates. Data are expressed as mean ± SD. f) Assessment of the mean indel efficiency of four cytosine base editors using the plot based on the data in Figure 1e. Mean indel frequency at each site is represented by individual data points, with the central dotted line indicating the overall mean. Two‐tailed paired t‐tests were performed: not significant (ns) *P* ≥0.05, * *P* < 0.05, ** *P* < 0.01, and *** *P* < 0.001.

To assess the efficacy of TadA‐derived cytosine base editors (CBEs) in converting cytosine (C) to thymine (T) in zebrafish, and to systematically compare their editing efficiency, we selected three representative CBE systems, TadCBEmax, TadCBEa, CBE‐T1.14 from independent research groups, which have the best activity in human cells and harbor distinct mutations in the TadA domain.^[^ ^18^, ^19^, ^20^ ^]^ Both TadCBEmax and TadCBEa share similar TadA mutations (E27A, V28G) in the loop region. TadCBEa utilizes an evolved TadA‐8e backbone carrying PANCE/PACE‐selected cytidine‐deaminase mutations, achieving the highest raw C‐to‐T editing yet, while omitting UGIs and thus exhibiting elevated indels. TadCBEmax uses the same TadA‐8e variant but appends a single C‐terminal UGI to curb uracil excision, yielding similarly high on‐target editing with <2% indels. CBE‐T1.14 incorporates a distinct set of active‐site TadA mutations for cytidine specificity and is fused with two UGIs, like other CBEs, such as BE4max, delivering robust, low‐off‐target C‐to‐T editing in human cells. To comprehensively capture their editing characteristics, we selected 10 endogenous target loci with varying sequence contexts. All CBE constructs were generated using zebrafish codon optimization (**Figure** **1**a) and contained N‐terminal and C‐terminal bipartite nuclear localization signals (bpNLS). To analyze base editing outcomes, we injected 2′‐O‐methyl‐3′‐phosphorothioate (MS)‐modified single guide RNAs (sgRNAs) into one‐cell stage zebrafish embryos, extracted genomic DNA at 48 h post‐fertilization (hpf), and performed next‐generation sequencing (NGS) following polymerase chain reaction (PCR) amplification of target regions. Our NGS data revealed that all three TadA‐derived CBE systems achieved effective C‐to‐T base editing in zebrafish, albeit with varying efficiency (Figure 1b). Across all ten target sites, TadCBEa exhibited the highest efficiency, with observed efficiencies ranging from 1.70% to 47.85% The state‐of‐the‐art editor AncBE4max exhibited relatively high activity only at specific TC motifs, which is consistent with data reported in cell‐based systems^[^ ^7^ ^]^ (Figure 1c,d). TadCBEa and CBE‐T1.14 were associated with elevated indel frequencies, whereas TadCBEmax—comparable to AncBE4max—retained a low indel rate, indicative of improved editing precision (Figure 1e,f). Collectively, we demonstrated that each TadA‐derived CBE variant has certain limitations, necessitating their optimization to achieve specific C‐to‐T editing with low indel levels and high on‐target activity.

### 2.2 Characterization of a New TadA‐Derived CBE with High Efficiency and Low Indel Frequency

**Figure 2:**
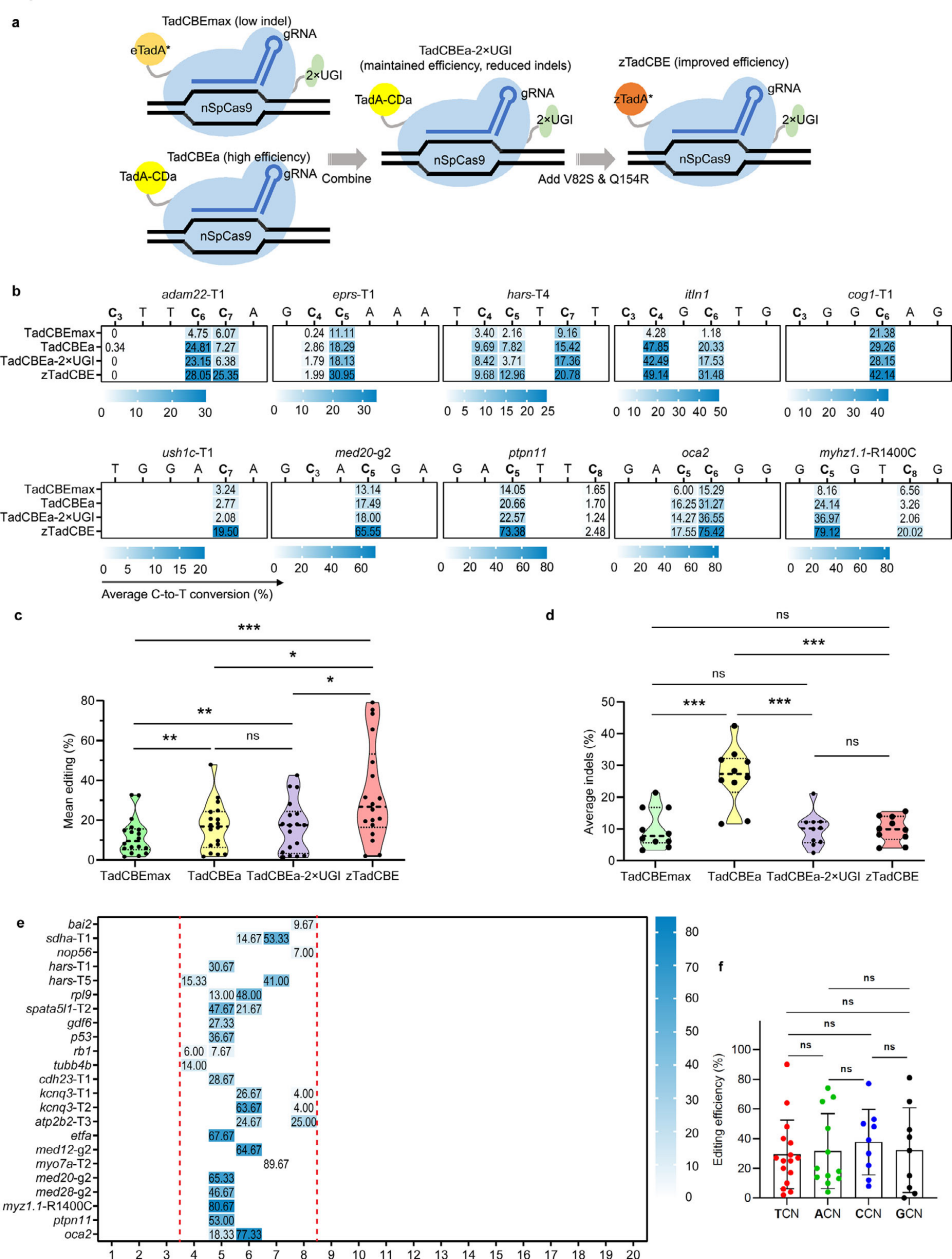
Efficient cytosine base editing mediated by zTadCBE in zebrafish. a) Schematic overview of the engineering strategy for zTadCBE. Starting from TadCBEmax (low indel), modifications were introduced sequentially: TadCBEa (increased efficiency), TadCBEa‐2xUGI (reduced indels while maintaining efficiency), and final zTadCBE, which incorporates additional mutations (V82S & Q154R) in the deaminase domain (eTadA*) for enhanced activity and editing precision. Spheres of different colors indicate deaminase variants with distinct mutations, nSpCas9 (D10A): SpCas9 nickase, UGI: Uracil glycosylase inhibitor. b) Heatmaps showing C‐to‐T base editing efficiencies among TadCBEmax, TadCBEa, TadCBEa‐2XUGI, and zTadCBE across ten target loci. Base position within the gRNA is denoted numerically, and values are reported as mean ± standard deviation (SD), with n = 3 biological replicates. Statistical analysis was conducted using a two‐tailed paired t‐test, not significant (ns) *P* ≥0.05, * *P* < 0.05, ** *P* < 0.01, and *** *P* < 0.001. c) Analysis of mean editing efficiency for TadCBEmax, TadCBEa, TadCBEa‐2XUGI, and zTadCBE based on data in Figure 2b. Mean editing efficiency per site is shown by individual data points, with the central dotted line representing the overall mean. Two‐tailed paired t‐tests were performed: not significant (ns) *P* ≥0.05, * *P* < 0.05, ** *P* < 0.01, and *** *P* < 0.001. d) Analysis of mean indel frequency for TadCBEmax, TadCBEa, TadCBEa‐2XUGI, and zTadCBE based on data in Figure 2b. Mean editing efficiency per site is shown by individual data points, with the central dotted line representing the overall mean. Two‐tailed paired t‐tests were performed: not significant (ns) *P* ≥0.05, * *P* < 0.05, ** *P* < 0.01, and *** *P* < 0.001. e) Heatmap illustrating the average C‐to‐T editing efficiency of zTadCBE across 23 target sites. Editing efficiency is shown on a color scale, where blue represents 100% efficiency and white represents 0% efficiency. f) Base editing efficiencies of the zTadCBE systems at the target C in different sequence contexts. Each data point reflects the mean editing activity at a specific site. Statistical analysis was conducted using a two‐tailed paired t‐test, not significant (ns) *P* ≥0.05, * *P* < 0.05, ** *P* < 0.01, and *** *P* < 0.001.

Given high on‐target activity of TadCBEa and low indel frequency of TadCBEmax, we hypothesized that a novel cytosine base editor (CBE) system could be constructed by integrating their advantageous features. Notably, TadCBEa lacks a UGI module, whereas TadCBEmax incorporates one (Figure S1, Supporting Information). Considering the role of UGI in suppressing uracil excision and undesired repair outcomes, we reasoned that incorporating UGI into TadCBEa might reduce indel formation while preserving its high on‐target activity. Accordingly, we constructed TadCBEa‐2xUGI by integrating 2X UGI modules into the TadCBEa scaffold. Subsequently, we evaluated its editing characteristics in zebrafish (**Figure** **2**a). High‐throughput sequencing of 10 endogenous loci in zebrafish revealed that TadCBEa‐2xUGI significantly reduced indel formation (from an average of 26.72% to 9.78%) while maintaining high on‐target editing efficiency comparable to TadCBEa (Figure 2b–d).

In addition to these efforts, we have recently developed an advanced TadA‐based adenine base editor (ABE) variant, ABE‐ultramax, which incorporates V82S and Q154R mutations in the deaminase domain.^[^ ^16^ ^]^ This variant achieved up to 100% efficiency in A‐to‐G editing in zebrafish.^[^ ^16^ ^]^ We hypothesized that these beneficial mutations might similarly enhance TadA‐derived CBE systems. Accordingly, we introduced the V82S and Q154R mutations into the TadA domain, creating a new CBE variant that we named zTadCBE (Figure 2a; Figure S2, Supporting Information). Comparative analyses across the ten target loci revealed that zTadCBE exhibited more than double the on‐target editing efficiency compared to TadCBEa‐2XUGI, with a maximum of 79.12% at the *myhz1.1*‐R1400C locus (Figure 2b,c). Furthermore, the indel frequency of zTadCBE was comparable to that of TadCBEa‐2xUGI, resulting in a significant reduction relative to TadCBEa (Figure 2d).

To comprehensively assess the performance of zTadCBE in zebrafish, we examined 23 additional endogenous targets. Editing activity, defined as the maximum observed activity at any position within each target site, ranged from 7.67% to 89.67% (Figure 2e). The primary editing window of zTadCBE spans five nucleotides (positions 4–8) (Figure 2e), consistent with the editing window of AncBE4max.^[^ ^8^ ^]^ We further observed that zTadCBE did not exhibit a pronounced sequence context bias (Figure 2f), although reduced activity was noted at specific AC or GC sites.

### 2.3 Expanding the Targeting Range and Editing Windows of TadA‐Derived CBEs

**Figure 3:**
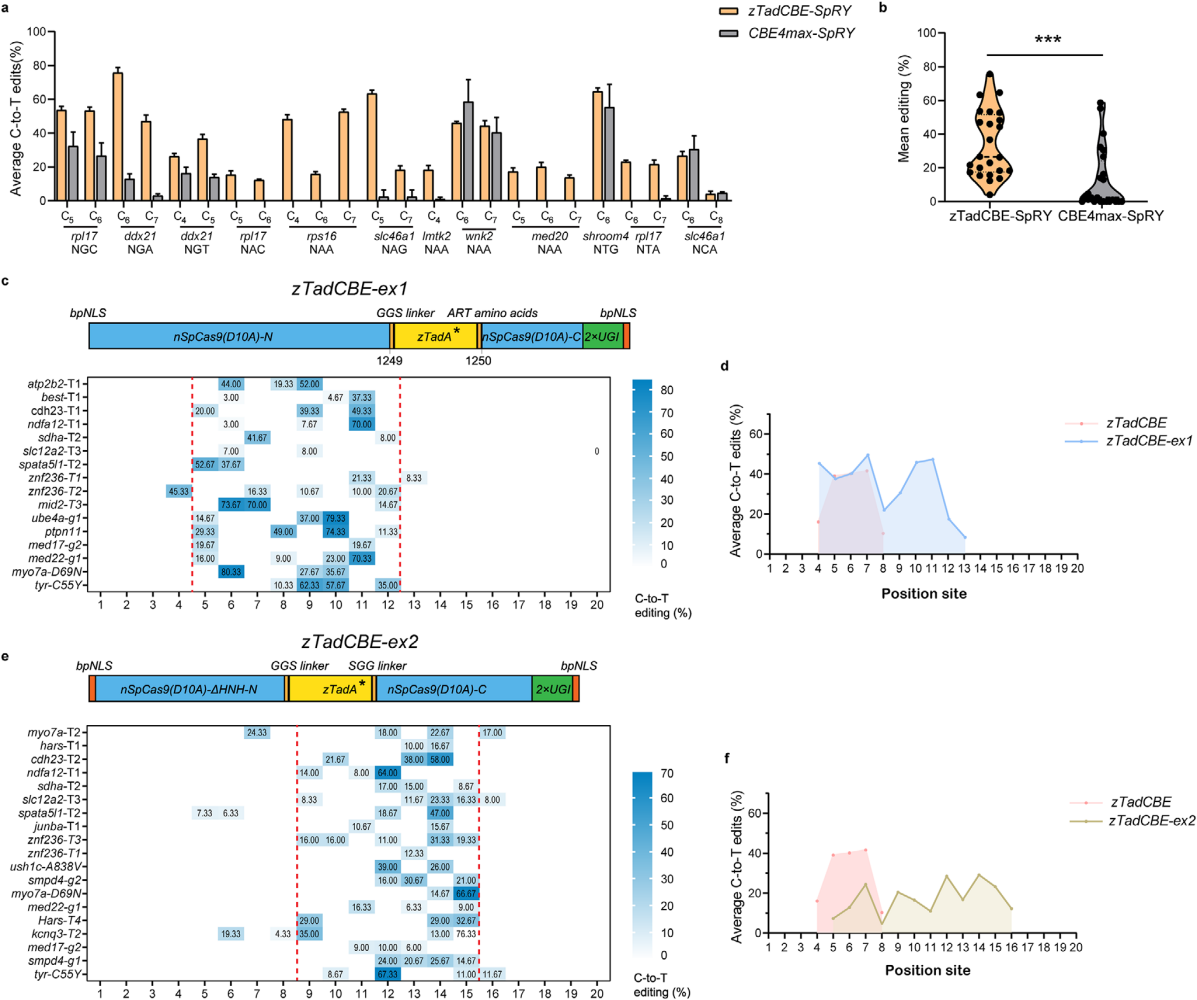
Broadening the Targeting Range and Editing Windows by zTadCBE variants. a) Comparison of average C‐to‐T editing efficiencies between zTadCBE‐SpRY and CBE4max‐SpRY using twelve gRNAs targeting NNN PAMs. zTadCBE‐SpRY shows consistently higher editing efficiencies at multiple non‐NGG PAM sites, demonstrating improved PAM flexibility and activity. The position of the edited base within each gRNA is indicated numerically. Data are presented as mean values ± standard deviation (SD), calculated from three biological replicates. b) Analysis of mean editing efficiency for zTadCBE‐SpRY and CBE4max‐SpRY based on data in Figure 3a. Mean editing efficiency per site is shown by individual data points, with the central dotted line representing the overall mean. Two‐tailed paired t‐tests were performed, with P values annotated at the top of the violin plot. c) Schematics showing constructs of zTadCBE‐ex1 designed to shift the editing window of cytosine base editing.Heatmap illustrating the average C‐to‐T editing efficiency of zTadCBE‐ex1 across 16 target sites. Editing efficiency is shown on a color scale, where blue represents 80% efficiency and white represents 0% efficiency. d) Comparison of editing efficiency and activity windows between zTadCBE and zTadCBE‐ex1. Each data point indicates the mean editing efficiency at a given target site. The editing windows, defined from the 5′ to 3′ end of the protospacer, are highlighted in pink (positions 4–8) for zTadCBE and in blue (positions 4–13) for zTadCBE‐ex1. Data were derived from three biologically independent replicates. e) Schematic representations of zTadCBE‐ex2 constructs. A heatmap depicting the average C‐to‐T editing efficiency of zTadCBE‐ex2 across 19 target sites is shown, with a color gradient indicating efficiency levels, where blue denotes 70% efficiency and white indicates 0% efficiency. f) Comparison of editing efficiency and activity windows between zTadCBE and zTadCBE‐ex2. Each data point indicates the mean editing efficiency at a given target site. The editing windows, defined from the 5′ to 3′ end of the protospacer, are highlighted in pink (positions 4–8) for zTadCBE and in yellow‐brown (positions 5–16) for zTadCBE‐ex2. Data were derived from three biologically independent replicates.

Base editors derived from the Cas9 protein of *Streptococcus pyogenes* (SpCas9) are limited by the NGG protospacer‐adjacent motif (PAM), restricting base editing to an editing window usually 4–8 bases distal to the PAM. Recent studies have demonstrated that the engineered SpCas9 variant, SpRY, with highly flexible PAM sequences, is compatible with single‐base editing systems in zebrafish, enabling the recognition of atypical NNN PAM sequences and thus expanding the targetable range of base editors to any desired site.^[^ ^3^, ^23^ ^]^ To broaden the targeting scope of zTadCBE‐mediated cytosine base editing, we substituted SpCas9n of the zTadCBE construct with SpRYnCas9, resulting in zTadCBE‐SpRY. To assess the C‐to‐T editing efficiency of zTadCBE‐SpRY at NNN PAM sites in zebrafish, we selected 12 target loci with atypical PAM sequences and injected single guide RNAs (sgRNAs) together with zTadCBE‐SpRY‐encoding mRNA into one‐cell stage embryos. Sequencing results showed that all 12 loci exhibited C‐to‐T conversion with editing efficiencies ranging from 4% to 75.67% across all sites (**Figure** **3**a). To minimize potential biases in comparison, we codon‐optimized CBE4max‐SpRY for expression in zebrafish. Compared to this optimized version—which is the most flexible cytosine base editor (CBE) reported thus far for targeting the zebrafish genome^[^ ^24^ ^]^—zTadCBE‐SpRY demonstrated an average six‐fold increase in C‐to‐T editing efficiency (Figure 3a,b). Importantly, zTadCBE‐SpRY could also effectively target specific loci inaccessible to CBE4max‐SpRY, such as *rpl18*‐NAC, *rps16‐*NAA, and *med20*‐NAA (Figure 3a). These findings indicate that TadA‐based C‐to‐T base editing is compatible with SpRYCas9n, supporting efficient editing by recognizing a highly versatile PAM range in zebrafish, and further expanding the targeting range.

While the PAMless variant substantially broadened the theoretical targeting range, the editing window of both zTadCBE‐SpRY and zTadCBE remains relatively narrow. This positional constraint of cytosine conversion within a defined window presents an inherent limitation to the flexibility of single‐base editing, regardless of PAM accessibility. We and others have previously shown that altering the relative positioning between the deaminase and Cas9 can shift the editable window of adenine base editors (ABEs).^[^ ^16^, ^25^, ^26^ ^]^ Whether this approach can also be applied effectively to TadA‐based CBEs has not yet been reported. To explore this possibility, we used the same positioning strategy to construct two novel CBE variants, zTadCBE‐ex1 and zTadCBE‐ex2 (Figure 3c,e). We tested their target windows using 16 and 19 target sites in zebrafish, respectively. Notably, on‐target editing activity of zTadCBE‐ex1 remained comparable to zTadCBE but with an expanded editing window spanning positions 4 to 13 of the protospacer (distal to the PAM), with peak activity concentrated between positions 5 and 12, compared to primary editing window of positions 4–8 of zTadCBE (Figure 3c,d). In contrast, zTadCBE‐ex2 exhibited slightly reduced on‐target activity compared to zTadCBE, but the editing window was markedly shifted toward the PAM‐distal region of the protospacer, spanning positions 5 to 16, with peak activity concentrated between positions 9 and 15 (Figure 3e,f).

Taken together, our findings demonstrated that the PAM‐flexible variant, zTadCBE‐SpRY, together with the zTadCBE‐ex1 and zTadCBE‐ex2 variants, provide high‐efficiency C‐to‐T editing in zebrafish with expanded targeting capabilities. This alleviates previous limitations of CBEs and allows access to previously inaccessible sites, offering greater flexibility in selecting efficient editors for diverse loci of interest. Our observation of consistently high (≈60%) germline targeting efficiencies, with transmission rates exceeding 70% for both zTadCBE and zTadCBE‐SpRY across four distinct loci (Table S1, Supporting Information), further confirms their ability to produce precise base edits and transmit these targeted edits to the germline with high efficiency.

### 2.4 Assessment of zTadCBE Off‐Target Effects in Zebrafish

**Figure 4:**
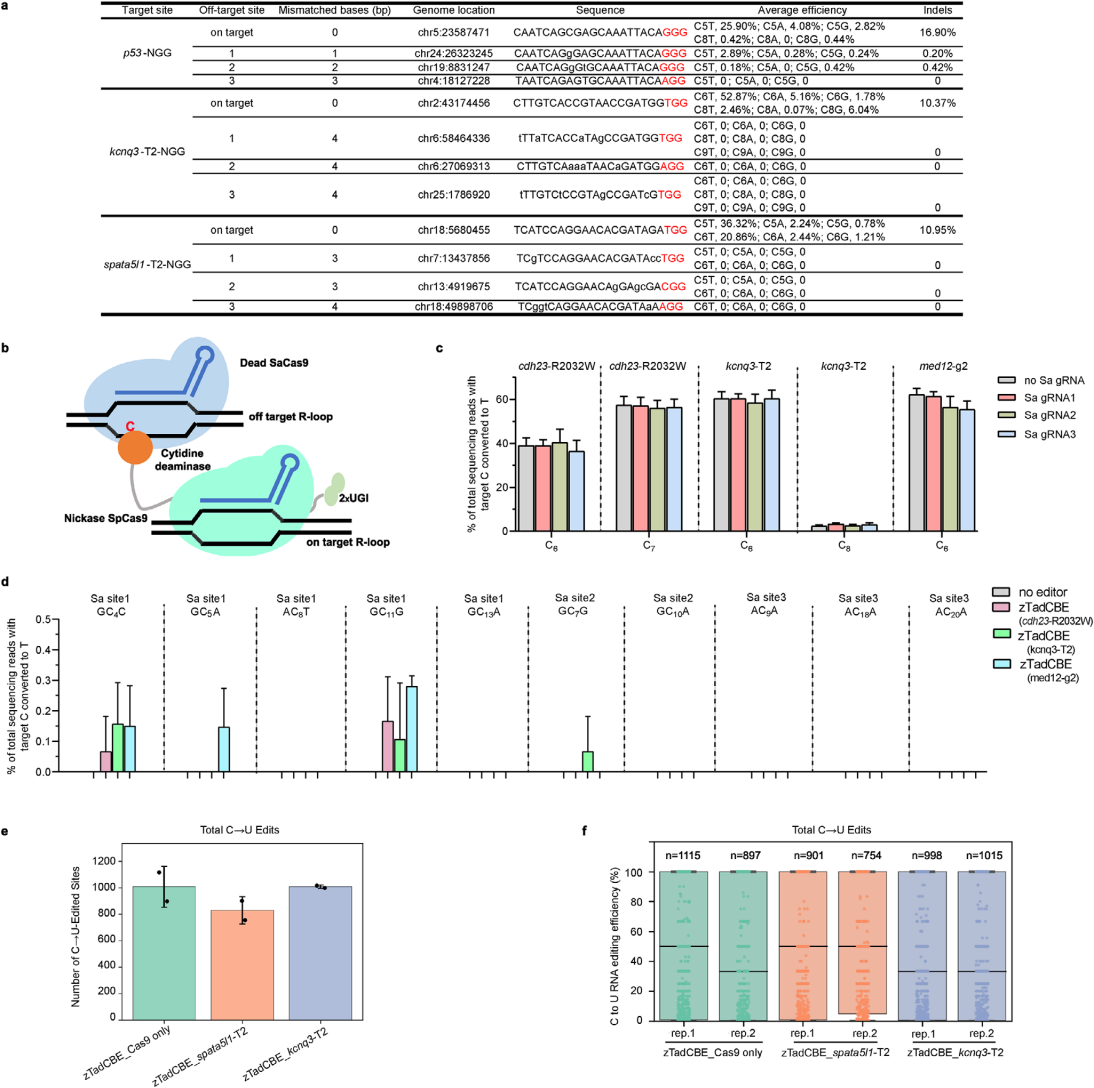
Off‐target analysis of zTadCBE in zebrafish. a) On‐target, product purity, and gRNA‐dependent off‐target analysis of zTadCBE induced C‐to‐T editing at *p53*, *kcnq3*‐T2, and *spata5l1*‐T2 sites using NGS. The top three high‐scoring off‐target sites (PAMs are underlined) are shown. Mismatched bases are indicated in lowercase. The PAM sequences are underlined in red. b) Schematic of Cas9‐independent deamination of cytosines within dSaCas9‐induced R‐loops by SpCas9 zTadCBE. c) Bar graphs depicting the efficiency of on‐target editing for different conditions, with heights representing the percentage of successful edits and colors indicating different targets, *cdh23‐R2032W*, *kcnq3‐T2*, and *med12‐g2*. Data are presented as mean values ± standard deviation (SD), calculated from three biological replicates. d) Bar graphs showing the editing efficiencies of three R‐loop regions. e) Transcriptome analysis of edited cytosine nucleotides in zebrafish embryos. Embryos were injected with zTadCBE+ *spata5l1*‐T2, zTadCBE + *kcnq3*‐T2 or zTadCBE mRNA only. Data from two independent replicates are shown. f) RNA C‐to‐U conversion frequencies in injected zebrafish embryos. The numbers of C‐to‐U RNA edits are indicated on the plots. Data from two independent replicates are shown.

Given the potential for high editing activity to increase off‐target effects, we systematically evaluated both Cas9‐dependent and Cas9‐independent off‐target activities of zTadCBE in zebrafish. For Cas9‐dependent off‐target assessment, we selected the top three predicted off‐target sites for each of the three target loci using the CRISPOR tool. These sites were PCR‐amplified and subjected to next‐generation sequencing (NGS). The analysis revealed minimal off‐target editing at these sites, with frequencies remaining below 3% (**Figure** **4**a). To investigate Cas9‐independent off‐target effects, we employed an orthogonal R‐loop assay.^[^ ^27^ ^]^ This involved co‐injecting zTadCBE mRNA with its corresponding gRNA, along with a catalytically inactive *Staphylococcus aureus* Cas9 (dSaCas9) and gRNAs targeting three distinct R‐loop sites. The dSaCas9 system facilitated the formation of R‐loops—single‐stranded DNA regions that could potentially serve as substrates for deamination (Figure 4b). NGS analysis of these sites demonstrated that zTadCBE maintained consistent on‐target editing efficiency regardless of dSaCas9 presence (Figure 4c). Importantly, off‐target editing at the R‐loop sites was negligible, with frequencies below 0.3% compared to controls (Figure 4d).

The deaminase domains of base editors can also target RNA nucleotides in addition to DNA nucleotides.^[^ ^28^ ^]^ To assess potential off‐target effects in cellular RNAs, we performed transcriptome‐wide RNA sequencing on zebrafish embryos injected with zTadCBE mRNA, with or without gRNAs targeting *spata5l1* or *kcnq3* loci. While the deaminase alone induced low levels of RNA off‐target edits (notably C‐to‐U conversions), the presence of zTadCBE with gRNAs did not significantly increase RNA off‐target activity compared to controls (Figure 4e,f). Collectively, these results indicate that zTadCBE exhibits high specificity in zebrafish, with minimal off‐target effects at both the DNA and RNA levels.

### 2.5 Disease Modeling in Zebrafish Using TadA‐Derived CBEs

**Figure 5:**
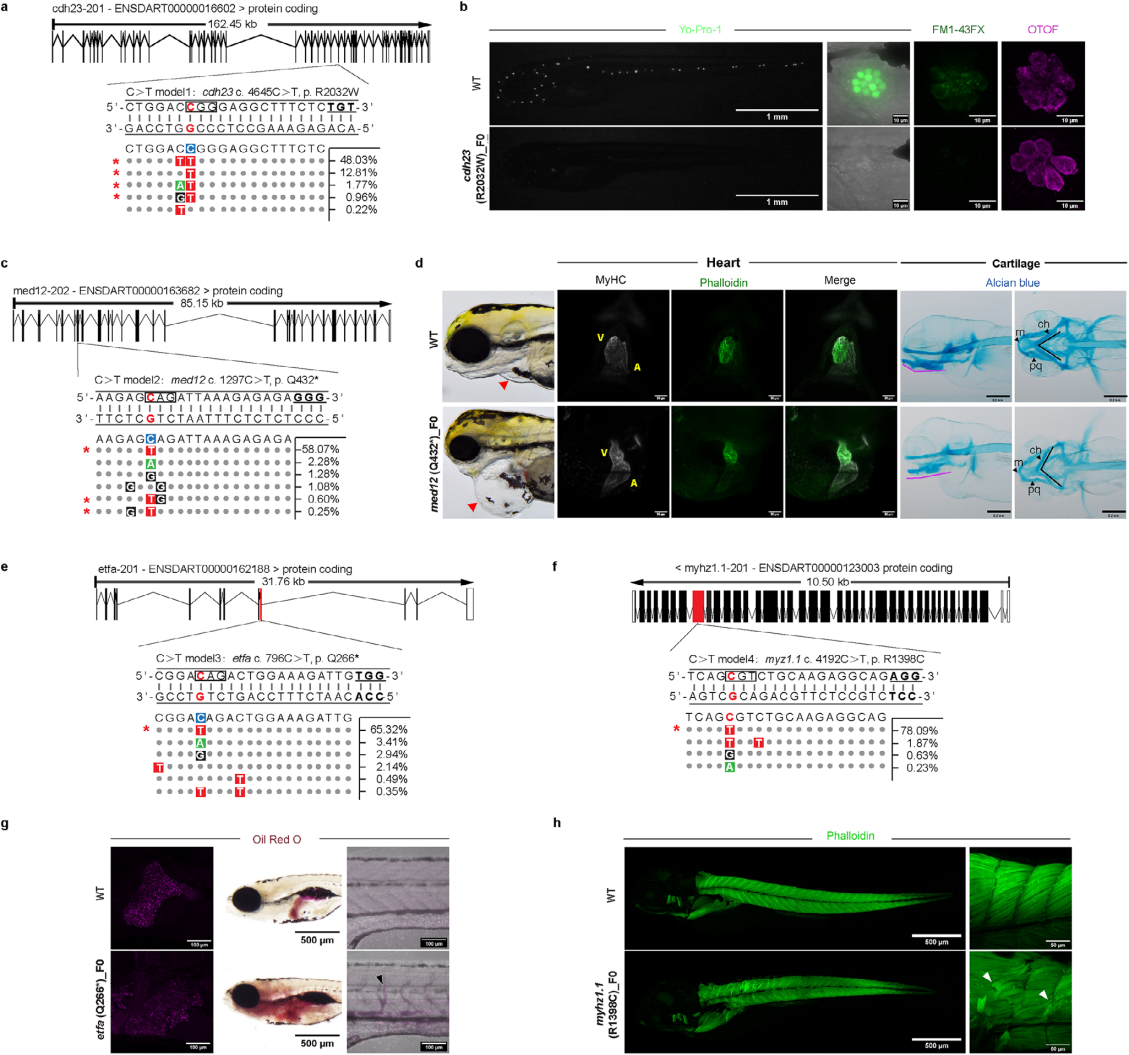
Disease modeling using zTadCBE editors. a) Schematic representation of CRISPR target site in the *cdh23* (R2032W) showing the genomic structure with exons (black bars) and introns in zebrafish. The targeted sequence is displayed with the PAM underlined and bold. The targeted nucleotide is highlighted in red. Next‐generation sequencing analysis of F0 zebrafish embryos injected with zTadCBE‐SpRY mRNA and gRNA targeting the *cdh23* (R2032W) locus. The predominant sequencing products with the highest frequencies are shown. The red asterisk indicates the desired edited product. b) Phenotypes of 5 dpf *cdh23* (R2032W) F0 embryos induced by zTadCBE‐SpRY. Compared to the control, F0 *cdh23* (R2032W) embryos exhibited a significant reduction in functional neuromast hair cells (labeled with YO‐PRO‐1 (green)). FM1‐43FX uptake was markedly reduced in hair cells of *cdh23* (R2032W) F0 embryos, indicating impaired mechanotransduction. OTOF immunostaining showed no appreciable difference between WT and mutant groups, suggesting preserved hair cell identity despite functional deficits. c) Schematic diagrams of *med12* (Q432*) in zebrafish. The targeted sequence is displayed with the PAM underlined and bold. The targeted nucleotide is highlighted in red, and a black bar indicates the related coding frame. Next‐generation sequencing analysis of F0 zebrafish embryos injected with zTadCBE mRNA and gRNA targeting the *med12* (Q4322*) locus. The predominant sequencing products with the highest frequencies are shown. The red asterisk indicates the desired edited product. d) Phenotypes of *med12* (Q432*) F0 embryos induced by zTadCBE. Compared to the control, *med12* (Q432*) F0 embryos exhibited microcephaly, microphthalmia, and pericardial edema (indicated by the red arrowhead) phenotypes. MyHC and phalloidin staining revealed that 3‐dpf *med12* (Q432*) F0 embryos exhibit abnormal heart chamber morphogenesis, implicating heart looping defects. Alcian blue staining further demonstrated craniofacial abnormalities in *med12* (Q432*) F0 embryos at 4 dpf. From the lateral view, the magenta line shows the angle and extent of Meckel's cartilage and palatoquadrate. In the ventral view, *med12*(Q432*) F0 embryos present a more expanded angle for ceratohyal, misaligned and shortened palatoquadrate, and kinked Meckel's cartilage. Overall, the craniofacial morphology of *med12*(Q432*) F0 embryos exhibits severe deformation. (ch: ceratohyal, pq: palatoquadrate, m: Meckel's cartilage). e) Schematic diagrams of *etfa* (Q266*) in zebrafish. The targeted sequence is displayed with the PAM underlined and bold. The targeted nucleotide is highlighted in red, the related coding frame is indicated by a black bar. Next‐generation sequencing analysis of F0 zebrafish embryos injected with zTadCBE mRNA and gRNA targeting the *etfa* (Q266*) locus. The predominant sequencing products with the highest frequencies are shown. The red asterisk indicates the desired edited product. f) Schematic diagrams of *myhz1.1* (R1398C) in zebrafish. The targeted sequence is displayed with the PAM underlined and bold. The targeted nucleotide is highlighted in red, and the related coding frame is indicated by a black bar. Next‐generation sequencing analysis of F0 zebrafish embryos injected with zTadCBE mRNA and gRNA targeting the *myhz1.1* (R1398C) locus. The predominant sequencing products with the highest frequencies are shown. The red asterisk indicates the desired edited product. g) Phenotypes of *etfa* (Q266*) F0 embryos induced by zTadCBE. Compared to the control, *etfa* (Q266*) F0 embryos exhibited global lipid accumulation when labelled with Oil Red O. In contrast, the deposition of lipid is restricted to the liver in the control. Fluorescent imaging further showed ectopic lipid deposition in vascular structures (indicated by black arrowhead). h) Phenotypes of 5 dpf *myhz1.1* (R1398C) F0 embryos induced by zTadCBE. Phalloidin staining of F‐actin revealed a disorganized myotome structure across somites in myhz1.1 *myhz1.1* (R1398C) F0 embryos, with defects most pronounced in the anterior trunk region compared to the control.

Next, we aimed to utilize zTadCBEs to generate disease‐related genetic variants in zebrafish, leveraging the unprecedented ability of cytosine base editors (CBEs) to introduce patient‐specific point mutations that mimic the genetic basis of many human diseases.^[^ ^29^ ^]^ First, we targeted a clinically relevant mutation in CDH23 (c.6085C>T; p.R2032W), a gene commonly associated with non‐syndromic prelingual hearing loss.^[^ ^30^ ^]^ Using zTadCBE‐SpRY, we edited the desired position (position 7 from the 5′ end) with an editing efficiency of 63.57% (based on pooled high‐throughput sequencing results from 10 embryos). Although this also resulted in editing the neighboring bystander cytosine at position 6 from the 5′ end, this led to a synonymous mutation that did not alter the amino acid sequence of *cdh23* (**Figure** **5**a). We investigated the impact of this mutation on hair cell development and function using YO‐PRO‐1, a live dye which enters hair cells via mechanoelectrical transduction (MET) channel and thus can be utilized as an assay for function of hair cells in the zebrafish lateral line.^[^ ^31^ ^]^ YO‐PRO‐1 labeling revealed diminished dye uptake for *cdh23* (R2032W) F0 embryos when compared to wild‐type (WT) controls. To confirm whether the diminished dye uptake was due to loss of hair cell function or development, we performed immunohistochemistry using hair cell marker otoferlin (OTOF, HCS‐1 antibody) and another vital dye FM1‐43FX, which specifically enters mechanotransducing hair cells via functional transduction channels to investigate the function and development of hair cells. We found that FM1‐43FX uptake was significantly reduced in the *cdh23*(R2032W), indicating impaired mechanotransduction activity. At the same time, HCS‐1 antibody staining showed no difference in the total number of hair cells (mechanotransducing plus non‐mechanotransducing hair cells) between WT and *cdh23* (R2032W) F0 groups. Together, these results suggest that only the mechanotransduction, but not the development, of hair cells is affected in *cdh23*(R2032W) (Figure 5b), consistent with previous results from *cdh23* knockout mutants.^[^ ^31^ ^]^

Next, we induced a point mutation in the *MED12* gene, which encodes Subunit 12 of the Mediator complex, a protein that regulates transcription by facilitating interactions between transcription factors and RNA polymerase II. Given its essential role, mutations in *MED12* are implicated in various developmental and neuropsychiatric disorders.^[^ ^32^ ^]^ CRISPR‐mediated introduction of premature STOP codons (CRISPR‐STOP) has recently proven to be an efficient and less disruptive alternative to traditional Cas9‐mediated gene knockout approaches. Instead of introducing frameshift‐inducing indels through double‐strand breaks, CRISPR‐STOP employs base editors to install premature stop codons at defined coding positions, enabling precise and predictable gene silencing.^[^ ^33^ ^]^ Using this strategy, we introduced a C‐to‐T transition at *med12* c.1297C>T (p.Q432*) to encode a premature stop codon located in a GC‐rich context. zTadCBE achieved efficient editing at this site, with a mean on‐target conversion rate of 58.67% based on pooled high‐throughput sequencing from 10 embryos. (Figure 5c). Phenotypic analysis of F0 embryos revealed several characteristic defects associated with *med12* loss‐of‐function,^[^ ^34^, ^35^ ^]^ including microcephaly, microphthalmia, and pericardial edema (Figure 5d). Further examination of 3 dpf larvae using Myosin heavy chain (MyHC, A4.1025 antibody) and phalloidin staining demonstrated abnormal heart chamber morphology, implicating defects in heart looping (Figure 5d). Alcian blue staining at 4 dpf further revealed craniofacial abnormalities (Figure 5d). Using antibodies against acetylated tubulin and synaptic vesicle glycoproteins SV2 to label the brain at 3 dpf, we found the injected embryos also exhibited misalignment of the midbrain and hindbrain, along with malformation of the forebrain ventricle and shorter anterior‐posterior extent of the brain (Figure S3, Supporting Information) These phenotypes are consistent with the wavy brain appearance, defective anterior‐posterior brain expansion, and failed forebrain ventricle formation previously reported in *med12*‐deficient zebrafish.^[^ ^36^ ^]^

Moving beyond neurodevelopmental disorders, we next used zTadCBE to generate a zebrafish model of a metabolic disorder by introducing a c.796C>T (p.Q266*) nonsense mutation, located within an AC motif, in the gene *etfa*, which encodes electron transfer flavoprotein subunit alpha, a mitochondrial protein involved in fatty acid and amino acid oxidation by transferring electrons to the respiratory chain (Figure 5e). We achieved highly efficient base conversion at the target site, with a mean editing efficiency of 65.32%. Phenotypic analysis of *etfa* (Q266*) F0 embryos revealed widespread lipid accumulation, in contrast to wild‐type controls: Oil Red O staining confirmed ectopic lipid deposition throughout the body, including in the vasculature (Figure 5g), where lipid buildup was visible along intersegmental vessels, which is consistent with the phenotype observed in our F0 knockout model.^[^ ^37^ ^]^

Finally, to examine a muscle‐related pathology, we introduced a C‐to‐T transition at *myhz1.1* c.4192C>T (p.R1398C), which encodes a myosin heavy chain protein specific to fast skeletal muscle in zebrafish, playing a key role in muscle contraction and development during embryogenesis using zTadCBE (Figure 5f). This site, located within a GC motif, was edited with a mean editing efficiency of 78.09%. Phalloidin staining of F‐actin in F0 embryos revealed disorganized myotome structure across somites, particularly in the anterior trunk region, compared to wild‐type controls (Figure 5h). These structural abnormalities suggest impaired muscle fiber patterning and differentiation resulting from the introduced mutation, reflecting similar phenotypic disruptions seen in our F0 knockout model.^[^ ^37^ ^]^

Collectively, our findings suggested that highly efficient CBEs are promising tools for rapid F0‐based functional gene analysis. In contrast, the state‐of‐the‐art editors ancBE4max (for NGG PAM) and CBE4max‐SpRY (for NNN PAM) performed poorly at these four loci, with editing efficiencies below 23% or entirely undetectable (Figure S4, Supporting Information). By comparison, zTadCBE and its variants achieved robust editing at the same sites, with efficiencies ranging from 58.67% to 78.09%, demonstrating marked advantages in both editing activity and target specificity. These properties make zTadCBE particularly well‐suited for constructing zebrafish disease models, greatly enhancing the precision and flexibility of genetic research in this area.

## 3 Discussion

Given the speed with which large numbers of genetic mutations are being identified, the development of CRISPR base editors has been a significant advancement in our ability to modify single nucleotides within the genome—particularly disease‐specific mutations—with high precision and efficiency. As such, base editors hold immense promise for studying disease‐specific variants in genetically tractable, complex model organisms such as mice and zebrafish.

Cytosine base editors (CBEs) based on APOBEC1 deaminases such as BE3 and BE4^[^ ^1^ ^]^ laid the groundwork for the continued development of base editors. However, their utility in animal models has been constrained by limited editing efficiency and sequence biases, particularly in zebrafish where TC‐context biases are more pronounced than in mammalian cells^[^ ^38^ ^]^, limiting their editing capabilities in AC, CC and GC contexts. While recent advancements, like AncBE4max, have improved the editing efficiency and accuracy of base editors, ^[^ ^8^ ^]^ which continue to exhibit sequence‐specific constraints and produce more indels in zebrafish compared to cell lines, thereby restricting which sites can be targeted and the purity of the intended edits.

In this study, we developed an optimized TadA‐derived CBE for zebrafish, zTadCBE, which addresses these limitations by significantly improving editing efficiency and sequence bias, minimizing off‐target edits, and expanding target site accessibility. We further demonstrated the potential of zTadCBE to substantially broaden the application of CBEs by targeting specific sequence contexts, successfully constructing four zebrafish disease models in the F0 generation. This demonstrates its potential for in vivo functional studies of pathogenic variants.

Despite the notable strengths of zTadCBE, certain limitations persist and should be addressed in future studies. Previous studies have reported that TadA‐derived CBEs also possess some A‐to‐G editing activity, leading us to further evaluate 20 loci for A‐to‐G conversions.^[^ ^18^, ^19^, ^20^ ^]^ Our results indicated that 4 out of these 20 sites exhibited minimal A‐to‐G activity (<10%) (Figure S5, Supporting Information), with most edited A bases situated within TA motifs, aligning with the intrinsic TA‐site preference of TadA. Although this low level of A‐to‐G activity is negligible in zebrafish applications, particularly since it can be diluted through germline transmission, it remains a concern for any potential clinical use of the tool. Therefore, further engineering is required to eliminate unintended A‐to‐G editing activity for therapeutic applications.

The second limitation of current TadCBE tools is their precision. Although the editing window of zTadCBE is generally restricted to positions 4–8, the system is still unable to effectively distinguish multiple editable Cs within this region. Therefore, the development of more refined base editors capable of targeting only one or two cytosines within a defined window is urgently needed. Several strategies have been proposed to enhance editing precision, including shortening the linker between Cas9 and the deaminase or introducing specific mutations into the deaminase to modulate its catalytic properties.^[^ ^39^, ^40^ ^]^ Nonetheless, it remains to be investigated whether these modifications can be effectively applied to zTadCBE.

The third major limitation lies in editing efficiency. Although we successfully generated four disease models in the F0 generation, the editing efficiencies at many loci remained below 50%, which poses a significant constraint for large‐scale functional screening of single‐nucleotide variants (SNVs). Therefore, the development of more broadly active and high‐efficiency CBE tools is critically needed. Further enhancement of zTadCBE to boost editing activity in F0 embryos could significantly accelerate the assessment of pathogenicity in SNVs, as demonstrated by the performance of our ABE‐Umax variant. Notably, recent work introduced next‐generation CBE6 variants through phage‐assisted evolution, achieving improved efficiency in mammalian systems.^[^ ^41^ ^]^ Future studies will explore whether such advanced CBEs can be adapted for use in zebrafish, with the potential to enable robust biallelic editing in the F0 generation and facilitate rapid in vivo functional validation.

During the revision of this manuscript, a study described a similar TadA‐derived tool, zTadA‐BE4max, which is reported to edit efficiently without sequence context bias in zebrafish.^[^ ^42^ ^]^ Notably, our construct features a different set of mutations within the TadA domain. The reanalysis of data revealed that while zTadA‐BE4max performs well at GC and CC motifs, its activity at ACN sites is limited. Comparing 18 endogenous zebrafish loci, including three (*twist2*‐g1, *ptpn11*, and *tuba1a*) poorly edited by zTadA‐BE4max, we found that zTadCBE and zTadA‐BE4max have comparable average editing efficiencies. However, zTadCBE significantly outperforms zTadA‐BE4max at ACN motifs, with no notable differences at CCN, GCN, or TCN sites, confirming zTadA‐BE4max's bias toward GCN > TCN ≥ CCN >> ACN motifs. Given the distinct engineering approaches of these tools, combining their beneficial mutations could yield a more versatile and efficient CBE for zebrafish, further advancing genome editing capabilities.

## 4 Experimental Section

### Ethical Statement

All zebrafish experiments were carried out as per protocols 24‐11, 24‐12, and 24–28 approved by the Institutional Animal Care Committee (IACUC) of Oklahoma Medical Research Foundation.

### Zebrafish Maintenance

Wildtype zebrafish strain NHGRI‐1^[^ ^43^ ^]^ were raised and maintained at 28.5 °C on a 14 h light/10 h dark cycle. The selection of mating pairs (12–15 months) was random from a pool of 30 males and 30 females. All experiments were conducted using wild‐type zebrafish.

### Construction of Plasmids

All variant plasmids were constructed based on the pT3TS‐zevoCDA1‐BE4max plasmid^[^ ^39^ ^]^ with respective modifications. For the AncBE4max, TadCBEmax, TadCBEa, CBE‐T1.14, and TadCBEa‐2XUGI plasmids, deaminase sequences were optimized to follow zebrafish codon preferences and were synthesized by GenScript. The optimized deaminase sequences replaced the original zevoCDA1 sequence. In the TadCBEa plasmid, the downstream 2X UGI sequence was removed. For the TadCBEmax construct, the GGS linker was substituted with a P2A sequence. For the zTadCBE plasmid, we introduced two mutations, V82S and Q154R, in the deaminase region of TadCBEa and added 2X UGI at the C‐terminus of Cas9. For zTadCBE‐SpRY, the SpRY sequence was cloned from the ABE‐Umax‐SpRY plasmid and replaced the SpCas9 component in zTadCBE. In constructing the zTadCBE‐ex1 plasmid, the zTadA* variant was inserted at the SpCas9 docking site at position 1249, using a GSSGSS linker and an ART amino acid linker to connect the N‐ and C‐termini of zTadA* with Cas9. For zTadCBE‐ex2, the HNH domain of Cas9 was removed, and a GGS linker was used to link SpCas9 S793 to the N‐terminus of zTadA*, with an SGG linker connecting the zTadA* C‐terminus to SpCas9 R919. All fusions and mutations were generated using the Vazyme Mut Express II Fast Mutagenesis Kit V2 (Cellagen Technology LLC, CA, USA). The assembled plasmids were then transformed and amplified in DH5α Chemically Competent Cells (New England Biolabs, MA, USA).

### Single Guide RNAs (sgRNAs) and mRNA Synthesis

All sgRNAs used in this study were chemically modified with 2′‐O‐methyl (M) and 2′‐O‐methyl 3′‐phosphorothioate (MS) modifications at both the 5′ and 3′ ends. They were synthesized by GenScript Inc. (New Jersey, USA) and Synthego Inc. (California, USA). Detailed target sequences are provided in Table S2 (Supporting Information). All mRNAs were transcribed in vitro from XbaI‐linearized templates (NEB, USA) using the T3 mMESSAGE mMACHINE Kit (ThermoFisher Inc., CA, USA) and purified with the Monarch RNA Cleanup Kit (New England Biolabs, MA, USA). The resulting capped mRNAs and synthetic sgRNAs were prepared in a 2000 ng µl^−1^ stock solution and stored at −80 °C for subsequent use.

### Embryo Microinjection, Morphological Phenotyping, and Imaging

A solution containing sgRNA at 200 ng µl^−1^ and Cas9 mRNA at 400 ng µl^−1^ was injected in a 2‐nl volume into one‐cell‐stage embryos. After 2 to 5 days post‐fertilization (dpf), the embryos were anesthetized in a 0.016% tricaine/MS‐222 solution (Sigma–Aldrich, MO, USA) and positioned in 3% methylcellulose (Sigma–Aldrich, MO, USA) for imaging.  Images were captured using an Olympus SZX12 stereomicroscope equipped with a DP71 color digital camera (Olympus, Japan). Following imaging, the embryos were genotyped to establish connections between their genotypes and observable phenotypes.

### Alcian Blue Staining

To visualize craniofacial cartilage development in 4‐dpf larvae, Alcian blue staining was performed using a modified protocol.^[^ ^44^ ^]^ Larvae were fixed overnight in 4% paraformaldehyde at 4 °C, then dehydrated through a graded ethanol series (20%, 50%, 70%) for 10 min each. Samples were stained overnight at room temperature in 0.4% Alcian blue (Sigma) dissolved in 70% ethanol containing 80 mm MgCl₂. After staining, the larvae were neutralized with saturated sodium tetraborate for at least 2 h, then washed with distilled water. They were subsequently bleached using a solution of 3% hydrogen peroxide and 1% KOH for 20 min. Following PBSTw washes, tissue clearing was performed in 1% trypsin for 1 h at room temperature. Finally, samples were rinsed in 0.25% KOH and cleared through a graded glycerol series before imaging.

### Oil Red O Staining

Oil Red O staining was conducted according to the protocol described earlier.^[^ ^37^ ^]^ Larvae at 8 dpf were fixed overnight at 4 °C in 4% paraformaldehyde, then washed three times with 0.1% Tween‐20 in PBS (PBSTw). Samples were incubated for 15 min at room temperature in a staining solution containing 0.5% Oil Red O (300 µL in 100% isopropanol) mixed with 200 µL of distilled water. After staining, larvae were washed three times with PBSTw, followed by two 5‐min rinses in 60% isopropanol. Post‐staining, the larvae were briefly rinsed in PBSTw, post‐fixed in 4% PFA for 10 min, and then mounted in 1.2% low‐melting agarose.

### Whole‐Mount Immunohistochemistry Staining of Larvae

For YO‐PRO‐1 staining, 5 days post‐fertilization (dpf) larvae were exposed to 2 µm YO‐PRO‐1 in E3 medium and then rinsed with E3 medium. Subsequently, the larvae were mounted in 1.2% low‐melting agarose on a 34 mm glass‐bottom culture dish and imaged using an epifluorescence microscope (Olympus SZX12) for whole larvae and a widefield microscope (Zeiss AxioObserver Z1/7) with a C‐Apochromat 40x/1.20 W Korr objective lens for neuromasts.

For immunohistochemistry of hair cell mechanotransduction (FM1‐43FX + OTOF, 5 dpf), larvae were exposed to 3 µm FM1‐43FX in E3 media for 45 s and then washed with E3 media for 15 min thrice. The larvae were then fixed with 4% v/v paraformaldehyde (PFA), washed with 0.2% Triton X‐100 in phosphate‐buffered saline (PBSTx), permeabilized with 1XPBS + 3% Triton X‐100, and incubated with primary antibody against OTOF (HCS‐1 antibody, DSHB) and secondary antibody (goat anti‐mouse IgG Alexa Fluor 647 antibody, Jackson ImmunoResearch Laboratories) at 4 °C overnight.

For heart (3 dpf) and trunk muscle (5 dpf), larvae were fixed in 4% PFA at the indicated developmental stages. Fixed samples were washed in phosphate‐buffered saline containing 0.1% Triton X‐100 (PBSTx), then permeabilized with 3% Triton X‐100 overnight at room temperature. This was followed by an overnight incubation at 4 °C with a primary antibody against myosin heavy chain (MyHC, A4.1025 antibody, DSHB). For secondary antibodies and fluorescent F‐actin probes, goat anti‐mouse IgG Alexa Fluor 647 antibody (Jackson ImmunoResearch) and Alexa Fluor 488‐conjugated Phalloidin (1:100 dilution, Invitrogen, Cat# A12379) were used. The samples were then mounted in 1.2% agarose and imaged using a Zeiss Lightsheet 7 (for heart samples, 20X objective) and a Zeiss LSM710 confocal microscope (for whole‐mount muscle samples, 10X objective).

For whole‐mount immunohistochemistry of 3‐dpf zebrafish larval brain, larvae were fixed and stained as previously described.^[^ ^45^ ^]^

### Base Editing Analysis

Genomic DNA was extracted from three pooled samples, each containing six randomly selected embryos. For disease modeling experiments, genomic DNA was extracted from pools of 10 embryos per group to assess the average editing efficiency. A targeted locus of 150–300 bp was amplified using HotStart Taq‐Plus DNA polymerase (Qiagen, USA) with primers listed in Table S2 (Supporting Information). The PCR products were purified using the DNA Clean & Concentrator‐5 kit (ZYMO, USA) for sequencing. For Sanger sequencing, the results were analyzed with EditR (v1.0.10).^[^ ^46^ ^]^ For Next‐Generation Sequencing (NGS), paired‐end sequencing was performed on the Illumina MiSeq platform, and the data were processed using CRISPResso2 software to assess editing outcomes.^[^ ^47^ ^]^

### DNA Off‐Target Analysis

To identify potential gRNA‐dependent off‐target effects, each guide RNA (gRNA) underwent an in silico off‐target prediction analysis using Cas‐OFFinder^[^ ^48^ ^]^ and CRISPOR (Version 4.99).^[^ ^49^ ^]^ The specificity score for each predicted off‐target site was calculated with CRISPOR. The top three off‐target sites with the highest specificity scores were then selected for further validation using next‐generation sequencing (NGS). To assess gRNA‐independent off‐target editing using orthogonal R‐loop assays, a two nL injection mixture was prepared containing 400 ng µL^−1^ zTadCBE mRNA, 300 ngµL^−1^ dSaCas9 mRNA, 200 ngµL^−1^ SpCas9 guide RNA, and 200 ngµL^−1^ SaCas9 guide RNA, and microinjected into one‐cell‐stage zebrafish embryos. The designated genomic loci were subsequently analyzed for off‐target effects via targeted amplicon sequencing using the Illumina NovaSeq 6000 platform.

### RNA‐Seq Experiments and Data Analysis

Total RNA was extracted from two independent batches of 40 whole embryos per group at 2 days post‐fertilization using standard TRIzol‐based protocols. mRNA was enriched with oligo(dT) beads, fragmented, and reverse‐transcribed into cDNA, followed by library preparation and sequencing on the Illumina HiSeq platform. Raw reads were processed with fastp (version 0.18.0) for adapter and quality trimming and aligned to the GRCz11 zebrafish genome using HISAT2. Variant calling was performed using GATK, and C‐to‐U substitutions with 10 or more supporting reads were identified to assess RNA off‐target effects. C‐to‐U editing events were quantified using REDItools v1.3.

### Statistical Analysis

Sample sizes were not predetermined through statistical methods; however, randomization was applied, and no data were excluded from analysis. Unless otherwise stated, each experiment was repeated at least three times, with sample sizes specified in the figure legends or the Source Data. Data are shown as the mean ± standard deviation (SD). Statistical analyses were performed using GraphPad Prism version 8.0.2 (GraphPad Software, USA). Statistical significance was defined as *p < 0.05, **p < 0.01, and ***p < 0.001. To compare base editing efficiencies between different groups, two‐tailed unpaired Student's t‐tests were conducted, while comparisons of mean editing efficiencies within groups were evaluated using two‐tailed paired Student's t‐tests or nonparametric Wilcoxon matched‐pairs signed rank tests.

## Conflict of Interest

The authors declare no conflict of interest.

## Author Contributions

W.Q. and G.K.V. conceived the project. W.Q. performed the experimental work and analyzed the results. S.L., Y.Z., K.H., C.P., and P.V. contributed to the experiments. K.B performed bioinformatics analysis. W.Q. and G.K.V. wrote the original draft. All other authors reviewed and edited the manuscript. G.K.V. acquired funding and supervised the study.

## Supporting information



Supporting Information

Supporting Information

Supporting Information

Supporting Information

## Data Availability

The data that support the findings of this study are available from the corresponding author upon reasonable request.
