# Supplementary material for: Rationally Designed TadA‐Derived Cytosine Editors Enable Context‐Independent Zebrafish Genome Editing
Source: Adv Sci (Weinh). 2025 Jul 20;12(39):e09800. doi: 10.1002/advs.202509800 (PMC12533148; doi:10.1002/advs.202509800)
Supplement: Supplementary file 1 — Supporting Information [file ADVS-12-e09800-s001.docx]

**Supplementary Information**

**RATIONALLY DESIGNED TADA-DERIVED CYTOSINE EDITORS ENABLE CONTEXT-INDEPENDENT ZEBRAFISH GENOME EDITING**

Wei Qin^1^, Sheng-Jia Lin^1^, Yu Zhang^1^, Kevin Huang^1^, Cassidy Petree^1^_,_ Kevin Boyd^2^ Pratishtha Varshney^1^, Gaurav K. Varshney^1*^

1. Genes & Human Disease Research Program, Oklahoma Medical Research Foundation, Oklahoma City, OK, USA.
2. Cell & Cancer Biology Research Program, Oklahoma Medical Research Foundation, Oklahoma City, OK, USA.

* To whom correspondence should be addressed.

E-mail: [gaurav-varshney@omrf.org](mailto:gaurav-varshney@omrf.org)

**Supplementary Figures**

**
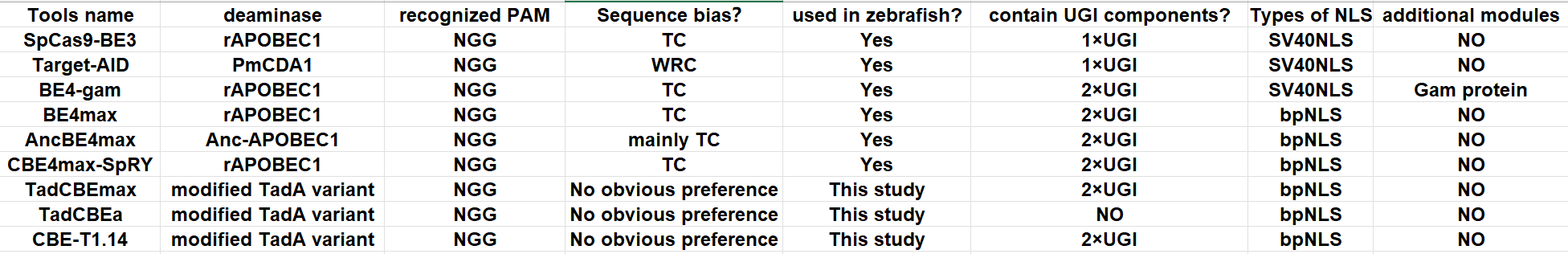
**

**Supplementary Figure 1. Summary of reported CBE variants with demonstrated activity in zebrafish and characteristics of TadA-derived CBE derivatives in this study.**

**
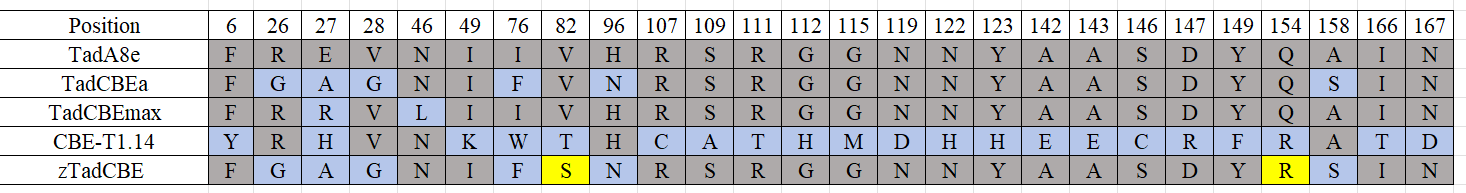
**

**Supplementary Figure 2. Mutation profiles of TadA deaminase domains in three representative CBEs in this study.** Mutations highlighted in gray are identical to those found in TadA8e, whereas blue indicates mutations unique to the three variants (TadCBEa， TadCBEmax, and CBE-T1.14) in comparison to TadA8e. Yellow marks mutations that are specific to zTadCBE relative to TadCBEa.

**
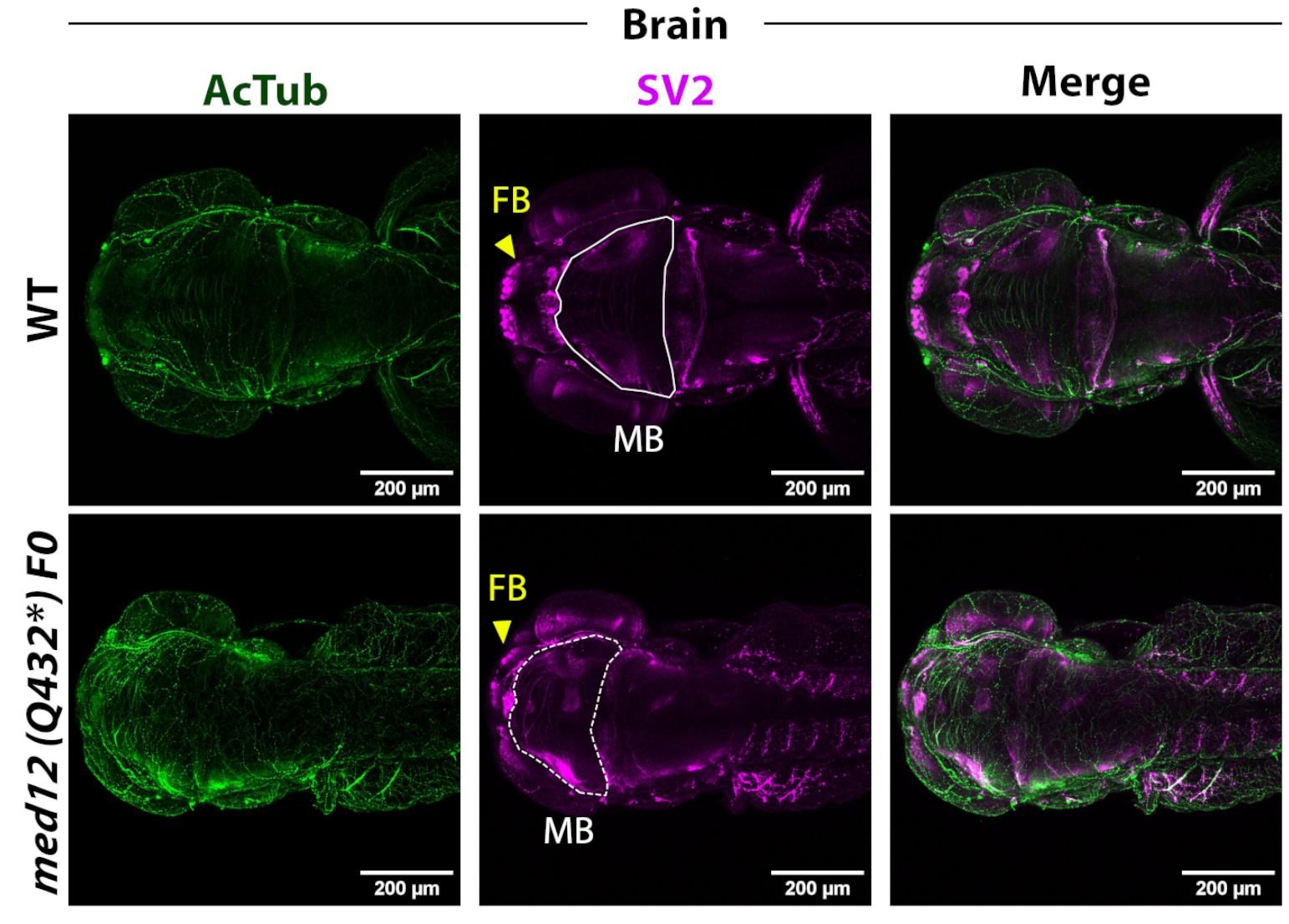
**

**Supplementary Figure 3. Characterization of brain developmental defects in *med12* (Q432*) F0 zebrafish embryos.** Whole-mount immunohistochemistry of *med12* (Q432*) F0 embryos at 3dpf exhibited abnormal brain development, characterized by shorter anterior-posterior brain extent, misalignment of the forebrain, midbrain, and hindbrain, as well as malformation of the forebrain ventricle. The white line (solid in WT and dashed in *med12*(Q432*) F0) in SV2 images were drawn to mark the midbrain, and the yellow arrow were used to depict forebrain. AcTub: Acetylated tubulin; SV2: synaptic vesicle glycoprotein 2; FB: forebrain; MB: midbrain.


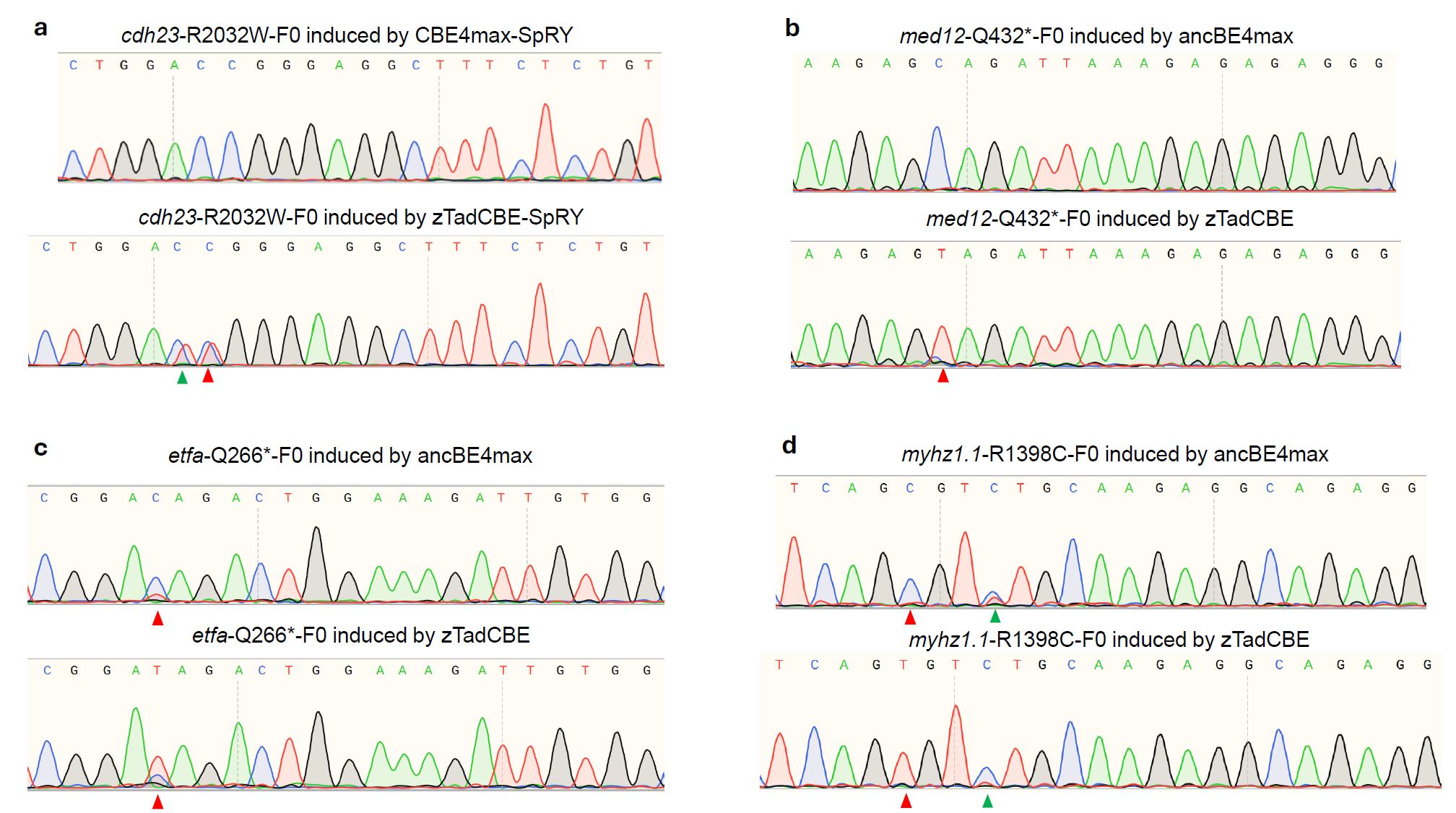


**Supplementary Figure 4. Performance of CBE4max-SpRY and AncBE4max Tools at Four Disease-Associated Loci**

1. Sanger sequencing results of *cdh23* (R2032W) F0 embryos induced by CBE4max-SpRY and zTadCBE-SpRY, respectively.
2. Sanger sequencing results of *med12* (Q432*) F0 embryos induced by AncBE4max and zTadCBE, respectively.
3. Sanger sequencing results of *etfa* (Q266*) F0 embryos induced by AncBE4max and zTadCBE, respectively.
4. Sanger sequencing results of *myhz1.1* (R1398C) F0 embryos induced by AncBE4max and zTadCBE, respectively.

The red arrowhead points to the expected nucleotide substitutions, while a green arrowhead in the Sanger sequencing chromatograms indicates bystander base substitutions.

**Supplementary Figure 5. The assessment of A-to-G activity of zTadCBE in zebrafish.** Evaluation the C-to-T and A-to-G activity of zTadCBE Efficiency in zebrafish. Each data point reflects the mean editing activity at a specific site. Data from three independent experiments were analyzed.

**
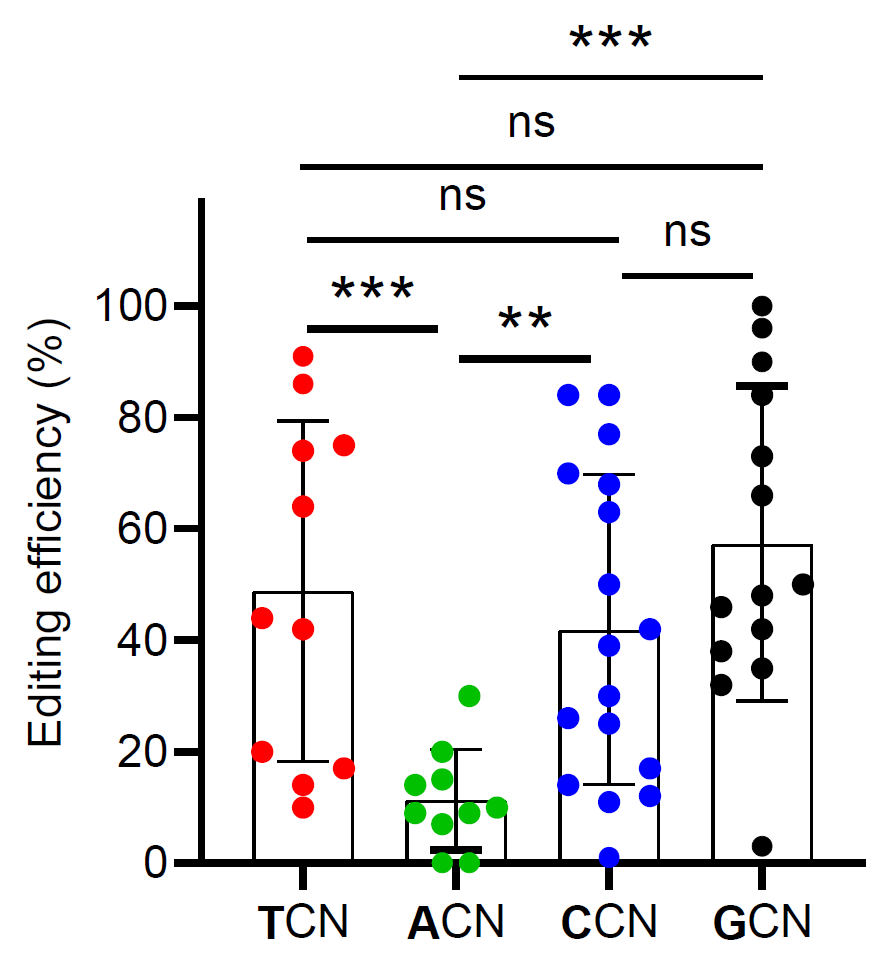
**

**Supplementary Figure 6. Activity Analysis of zTadA-BE4max Systems Across Different Sequence Motifs.** Base editing efficiencies of the zTadA-BE4max systems at the target C in different sequence contexts based on published data. Each data point reflects the mean editing activity at a specific site. Statistical analysis was conducted using a two-tailed paired t-test, not significant (ns) *P* ≥0.05, * *P* < 0.05, ** *P* < 0.01, and *** *P* < 0.001.


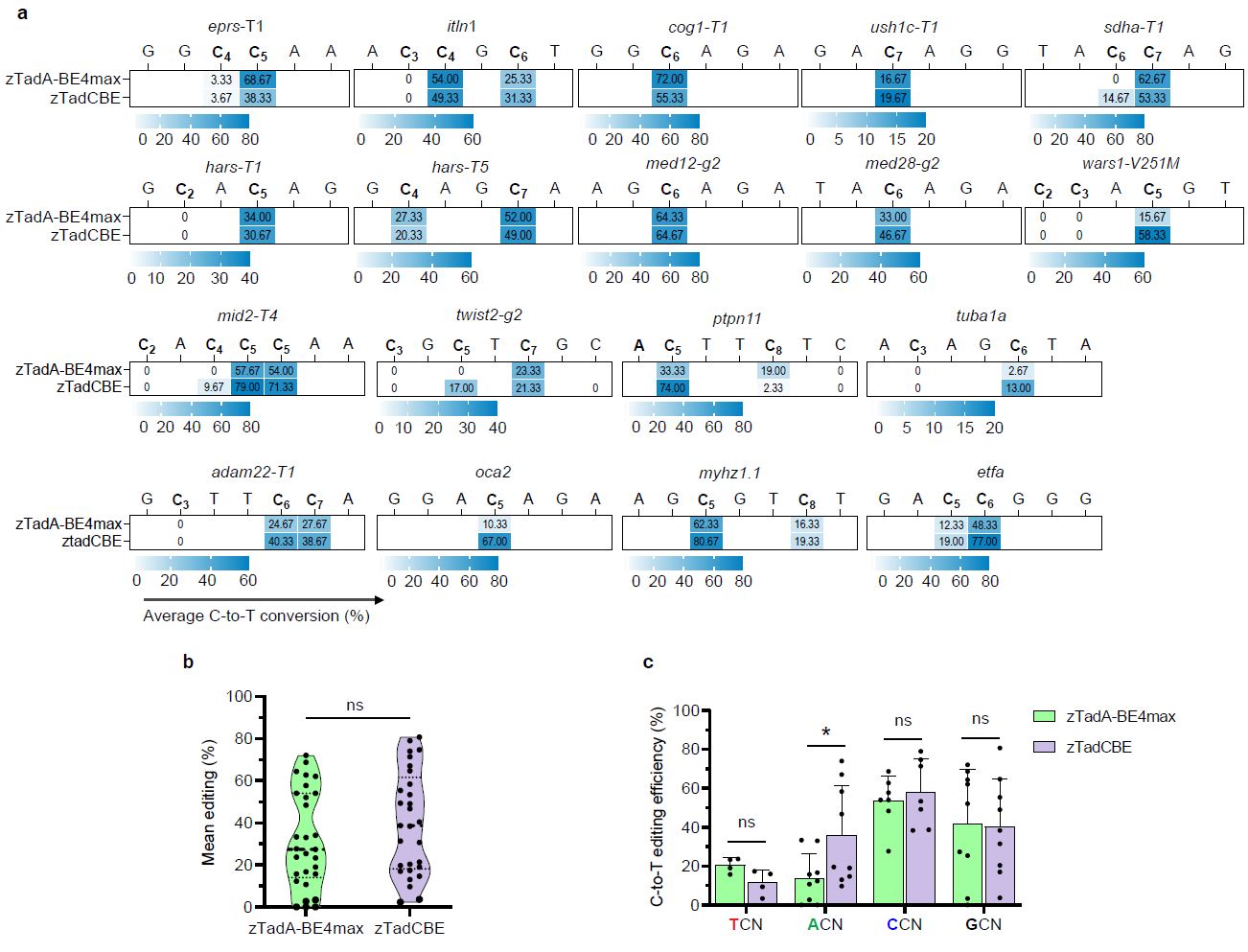


**Supplementary Figure 7. Comparative analysis of zTadA-BE4max and zTadCBE activities at identical genomic sites in zebrafish**

(**a)** Comparison of editing efficiency between zTadA-BE4max and zTadCBE across 18 target loci. The base position within the gRNA is denoted numerically, and values are reported as the mean ± standard deviation (SD), with n = 3 biological replicates.

(**b)** Analysis of mean editing efficiency for zTadA-BE4max and zTadCBE based on data in Fig. S8a. Mean editing efficiency per site is shown by individual data points, with the central dotted line representing the overall mean. Two-tailed paired t-tests were performed: not significant (ns) *P* ≥0.05, * *P* < 0.05, ** *P* < 0.01, and *** *P* < 0.001.

(**c)** Comparison of editing efficiency for zTadA-BE4max and zTadCBE across various NCN contexts.
